# Cancer survival in Lower Silesia: insights from a national oncology network pilot in Poland

**DOI:** 10.3389/fonc.2026.1739198

**Published:** 2026-02-18

**Authors:** Magdalena Władysiuk, Paweł Zawadzki, Paweł Prędkiewicz, Robert Plisko, Dawid Błaszczyk, Rafał Matkowski, Adam Maciejczyk, Bożena Cybulska-Stopa

**Affiliations:** 1 Collegium Medicum, Chair of Epidemiology and Preventive Medicine, Jagiellonian University, Krakow, Poland; 2 Lower Silesian Oncology, Pulmonology and Hematology Center, Wrocław, Poland; 3 Department of Finance, Wroclaw University of Economics and Business, Wrocław, Poland; 4 HTA Consulting, Krakow, Poland; 5 Department of Oncology, Wrocław Medical University, Wrocław, Poland; 6 Department of Hematology and Oncology, Faculty of Medicine, Wroclaw University of Science and Technology, Wrocław, Poland

**Keywords:** biological subtype, breast cancer, national oncology network, outcomes, Poland, stage, survival

## Abstract

**Introduction:**

This study provides population-based data on biological subtypes of breast cancer (BC) and associated survival outcomes among Polish women diagnosed between 2019 and 2023 in the Lower Silesia region during the national pilot phase of the National Oncology Network (NON).

**Methods:**

Data on BC cases were obtained from the DCOPIH databases as the leader of the pilot program in Poland in between 2019 and 2023 also in the Lower Silesia region. The main outcome was overall cancer survival and cases were linked to existing mortality databases. All patient consent and scope of data analysis was provided based on the MoH Regulation. Analyses included stage at diagnosis, biological subtypes, and five-year survival, stratified by age, subtype, and stage.

**Results:**

A total of 4490 women with BC were included. The luminal B subtype was the most prevalent, generally increasing with age and peaking at 46% in the 80+ age group. Across all stages, five-year survival was highest for luminal A (Kaplan Meier estimation of 90.3%. 95% CI: 88.1%–92.5%) and lowest for triple negative breast cancer (TNBC) (Kaplan Meier estimation of 68.5%, 95% CI: 63.4%-74.0%). For stages I disease, five-year survival was 92.8% for all subtypes aggregated (95% CI: 91.1%-94.5%). Survival declined with advancing stage, particularly for TNBC cases, in population with TNBC diagnosed at stage IV no patient surviving above 48 months was observed.

**Conclusions:**

Distinct clinical trajectories of BC subtypes have significant implications for prognosis and healthcare resource allocation. The observation of ≥92% five-year survival for stage I disease across subtypes underscores the critical importance of early detection, particularly in biologically aggressive subtypes such as TNBC breast cancer.

## Introduction

Breast cancer remains one of the most significant challenges in oncology and is the fourth most common cause of cancer-related deaths overall, ranking first among causes of cancer mortality in women (1). In Poland, it is the third most frequently diagnosed cancer, with 24, 418 new cases reported in 2022, accounting for 11.7% of all cancer diagnoses nationwide. The disease contributes substantially to cancer mortality and prevalence, with 8, 723 deaths (7.3% of all cancer-related deaths) and a five-year prevalence of 100, 017 cases, reflecting a large and growing population of women living with breast cancer or its sequalae (2).

Although breast cancer is increasingly recognized as a chronic disease, survival rates in Poland remain lower than the European average, despite significant global improvements over recent decades resulting from advances in early detection, treatment strategies, and public awareness. Since the mid-2000s, several initiatives have been undertaken in Poland to improve cancer care outcomes. These include two editions of the National Program for Cancer Disease Control. A key policy milestone occurred in January 2015, when a dedicated “fast-track” oncology pathway was introduced to expedite diagnosis and treatment for patients suspected of having cancer. In 2019, the National Oncology Network (NON) was piloted and now constitutes a central component of the National Cancer Strategy for 2020–2030 (3). A deeper understanding of the biological profiles of breast cancer within the Polish population is essential, as these features guide precision medicine approaches and provide prognostic and predictive value regarding treatment response, recurrence risk, metastatic patterns, and overall outcomes.

This article, for the first time, explores the breast cancer survival - encompassing tumor biology and patient characteristics—in women newly diagnosed between 2019 and 2023 in the Lower Silesia region, as part of the pilot implementation of the National Oncology Network.

## Methods

This study is a retrospective data analysis based on a cohort derived from the pilot implementation of the National Oncology Network (NON) in the Lower Silesia region, coordinated by the Lower Silesian Oncology Center (DCOPiH), covering the period from February 1, 2019, to March 31, 2023. Data were collected by the National Health Fund in cooperation with healthcare providers based on the respective ACT (4) and the MoH Regulations. The security and protection of data was assured. A total of 6, 917 female patients newly diagnosed with primary breast cancer were included. Data collection occurred in multiple stages. In the initial phase, patients who provided informed consent (in NON pilot) and had histopathologically confirmed diagnoses were enrolled. This retrospective cohort study followed the Strengthening the Reporting of Observational Studies in Epidemiology (STROBE) reporting guidelines.

Eligible participants were adult women (aged ≥18 years) with newly diagnosed primary breast cancer diagnosis (stage 0-IV) between from February 1, 2019, to March 31, 2023, regardless of treatment intent. Clinical data were extracted from medical records and coded according to the International Classification of Diseases for Oncology, Third Edition (ICD-O-3) (5), including invasive carcinoma (code C50) and *in situ* lesions (code D05). All patients included in the National Cancer Network (NCN) pilot program in Poland made a written declaration of informed consent to participate in the NCN pilot program (6). Before enrollment, each patient obtained information about the principles of the pilot program and the detailed information of personal data processing as part of the NCN (6) and the long effectiveness evaluation. Tumor staging was classified into five categories (0, I, II, III, and IV), and TNM staging was determined based on the AICC/UICC and the ESMO Clinical Practice Guidelines (7). Deaths from any cause were analyzed and survival curves were generated for the entire cohort, stratified by clinical stage and biological subtype, including luminal A, luminal B, HER2-positive, and triple-negative breast cancer.

Nominal variables were summarized using frequencies and percentages, while interval-scaled variables were described using the mean, standard deviation, median, range, and interquartile range (IQR). A statistical significance level of 0.05 was adopted for the analysis. Survival analysis was conducted using the Kaplan–Meier method for the entire cohort, as well as stratified by biological subtype, clinical stage, stage within subtype, and screening status. A complete-case approach was applied, in which follow-up time was calculated from the date of diagnosis to either the date of death or the end of the study period. Differences in survival between subgroups were assessed using the Cox proportional hazards model.

Five-year (60-month) overall survival was estimated using the Kaplan–Meier product-limit method. Because the diagnostic period (2019–2023) includes patients without the possibility of complete 5-year follow-up (e.g., those diagnosed in 2022–2023), the 60-month survival probability represents a Kaplan–Meier estimate at *t* = 60 months, i.e., an estimate obtained under right censoring rather than observed 5-year follow-up for all individuals. Patients contributed risk time from diagnosis until death or administrative censoring; those without an event by the censoring date were treated as right-censored.

Follow-up time was defined as the time from diagnosis to removal from the risk set (death or censoring). In the analytical cohort, follow-up ranged from 7 to 2, 269 days (median 1, 313 days, IQR 935–1, 723). Among censored individuals, time to censoring ranged from 35 to 2, 269 days (median 1, 388 days, IQR 1, 044–1, 790). Thus, the minimum potential follow-up in the dataset was 7 days (overall) and 35 days among those censored.

## Results

### Population

A total of 6, 917 women were included in the study cohort; however, the analytical dataset comprised 4, 490 patients (**Table 1**).

**Table 1:** Characteristics of women diagnosed with breast cancer in Lower Silesia participating in the pilot phase of the national oncology network.

Parameter	Total (%)
N	4490 (100%)
Gender	
Woman	4490 (100%)
Age	
Mean (SD)	61.6 (13, 2)
Median IQR range	63 (52-70) 22-99
Cancer stages (%)	
0	119 (2.7%)
I	1617 (36.0%)
II	1455 (32.4%)
III	983 (21.9%)
IV	316 (7.0%)
Type of breast cancer	
Luminal A	1363 (30.4%)
Luminal B	1917 (42.7%)
HER2+	801 (17.8%)
Triple negative	409 (9.1%)

A total of 6, 917 women who initiated the diagnostic pathway during the PSO program and for whom follow-up data were available for Kaplan–Meier analyses were initially eligible. The analytical cohort was derived using a complete-case approach for key tumour characteristics required for stratified and adjusted analyses (stage at diagnosis and biological subtype). We first excluded 418 women with missing stage information; within this group, 340 also had missing subtype data. From the remaining 6, 499 women with known stage, we additionally excluded 2, 009 women with missing subtype. The final analytical dataset therefore comprised 4, 490 women (overall exclusion: 2, 427/6, 917; 35.1%), with exclusions attributable to missing stage and/or subtype information.

To evaluate potential selection bias related to these exclusions, we compared age distributions between included and excluded individuals. Age was highly similar in the analytical cohort versus excluded cases (mean 61.6 vs 61.2 years; median 63.1 vs 62.8 years; SD 13.1 vs 13.5; range 22.4–98.8 vs 21.0–95.4), indicating negligible differences by this key baseline characteristic (standardized mean difference ≈0.03). The Wilcoxon rank sum test did not confirm the significance of median differences (p=0.30), and the Student’s t-test did not show any differences in means (p=0.25).

The mean age at breast cancer diagnosis was 61.6 years (SD = 13.1), with half of the patients being older than 63 years. The youngest patient was 22 years old, and the oldest was 99. Women aged 50–69 years—eligible for population-based breast cancer screening—constituted 53.9% of the sample.

Stage I was the most commonly diagnosed stage, accounting for 36.0% of cases, whereas stage IV disease was observed in 7.0% of patients. The most prevalent biological subtype was luminal B, identified in 42.7% of cases.

The percentage of patients diagnosed at stage 0 decreased from 3.4% in 2019 to 0.5% in 2023. The percentage of patients diagnosed at stage I in 2019–2022 varied in relatively narrow range from 34.5% in 2020 to 36.7% in 2021, while in 2023 the percentage was noticeably higher: 41.2%.

Throughout the observation period, the percentage of patients diagnosed at advanced stages (stage III or IV) ranged from 27.8% in 2019, to 29.9% in both 2020 and 2022, 28.6% in 2021, and declined to 27.3% in 2023.

In the analysed cohort, the age distribution within each biological subtype of breast cancer revealed significant differences (chi-squared test, p<0.001). Among patients with triple-negative breast cancer (TNBC), 12.2% were aged 19–39 years, indicating that younger women constituted a relatively larger share of this subgroup compared to other subtypes. Similarly, patients with HER2-positive tumours were more frequently observed in younger age categories (10.0% aged 19–39 years), whereas the proportion of young patients was markedly lower in the luminal A group (1.9%).

The luminal A subtype showed a pronounced shift toward older age groups, with 39.3% of patients aged 60–69 years and 7.9% aged ≥80 years. A similar trend, though slightly less pronounced, was observed for the luminal B subtype. The details of age and biological sub-type distribution are presented in **Table 2**.

**Table 2:** Age distribution (%) within biological subtypes of breast cancer in the study cohort.

Age	Luminal A (n=1363)	Luminal B (n=1917)	HER2-positive (n=801)	Triple-negative (n=409)	All (n=4490)
19-39	1.9%	5.9%	10.0%	12.2%	6.0%
40-49	14.3%	14.5%	18.5%	13.0%	15.0%
50-59	18.9%	18.9%	21.0%	22.2%	19.6%
60-69	39.3%	33.9%	27.6%	30.6%	34.1%
70-79	17.6%	18.2%	15.9%	15.4%	17.3%
80+	7.9%	8.7%	7.1%	6.6%	8.0%

### Kaplan-Meier survival estimations

The overall survival rate at 36 months from the time of diagnosis was 88.8% (95% CI: 87.8%-89.7%) (Kaplan-Meier extrapolation). During the first 12 months, survival was approximately 96.3% (95% CI: 95.8%-96.9%), decreasing to 92.0% (95% CI: 91.2%-92.8%) at 24 months. At 5 years Kaplan-Meier extrapolated overall survival rate was 82.6% (95% CI: 81.2%-84.0%). A DiLO (fast-track oncology) card was issued for 98.4% of patients (n = 4, 419), while 3.2% of patients did not initiate treatment within the Lower Silesia region.

The median time from the initiation of diagnostic procedures to the commencement of treatment was 66 days across all age groups. Any type of treatment was initiated within 49 days (7 weeks) in 37.5% of patients.

**Figure 1:**
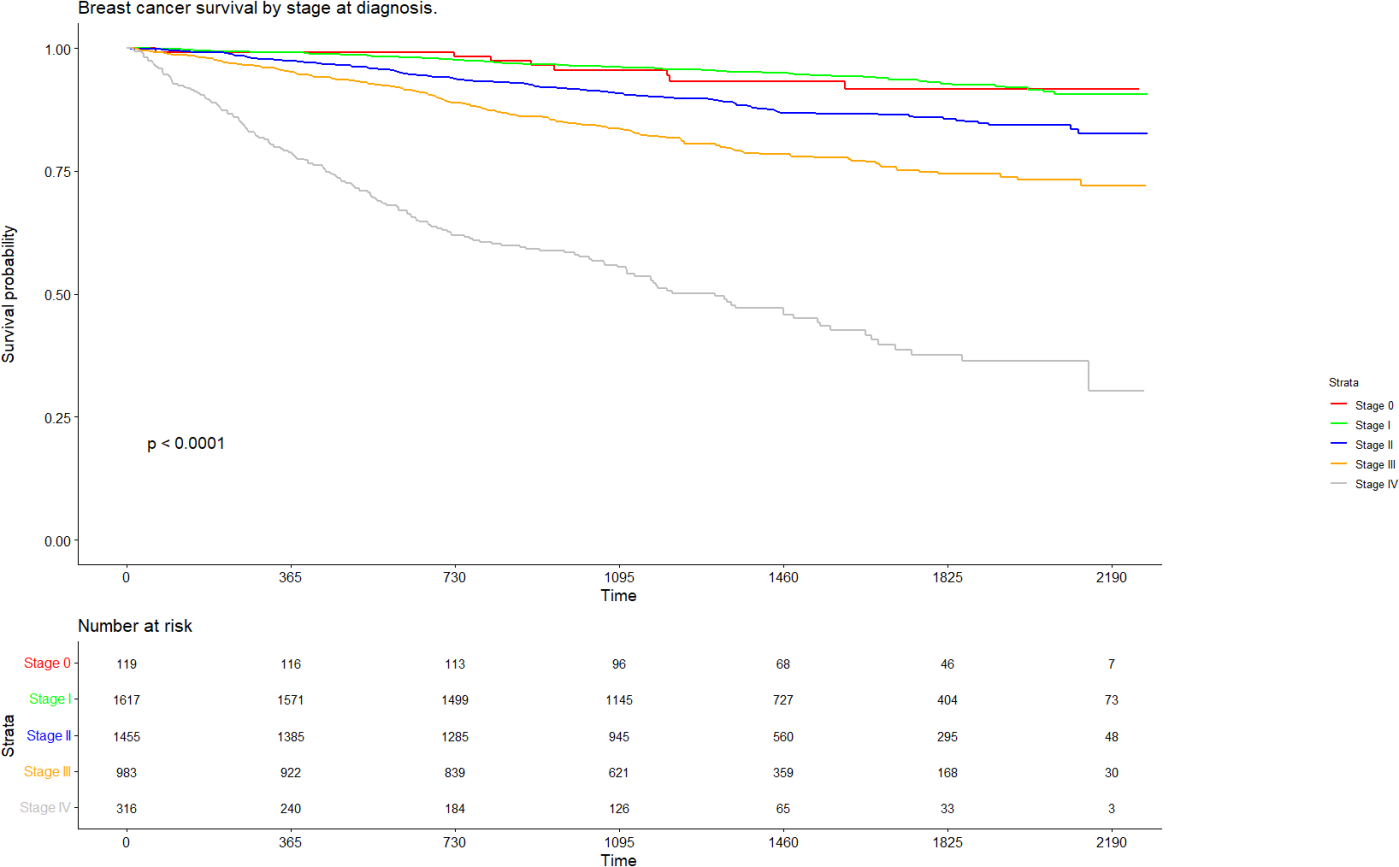
Breast cancer survival by stage at diagnosis.

In patients diagnosed at stage 0, Kaplan-Meier survival at 36/48/60 months was 95.6% (95% CI: 91.9%–99.4%), 93.3% (95% CI: 88.6%–98.3%) and 91.6%(95% CI: 86.1%–97.6%) respectively. In patients diagnosed at stage I, Kaplan-Meier survival at 36/48/60 months was therefore 96.1% (95% CI: 95.2%–97.1%), 94.9% (95%CI: 93.8%–96.1%) and 92.8% (95%CI: 91.1%–94.5%, respectively. In contrast, stage IV disease was associated with significantly lower survival – the extrapolated values were 55.4% (95% CI: 50.0%–61.4%) at 36 months, 45.7% at 48 months (95% CI: 39.8%–52.4%) and 37.6% (95% CI: 31.1%–45.4%) at 60 months (**Figure 1**).

**Figure 2:**
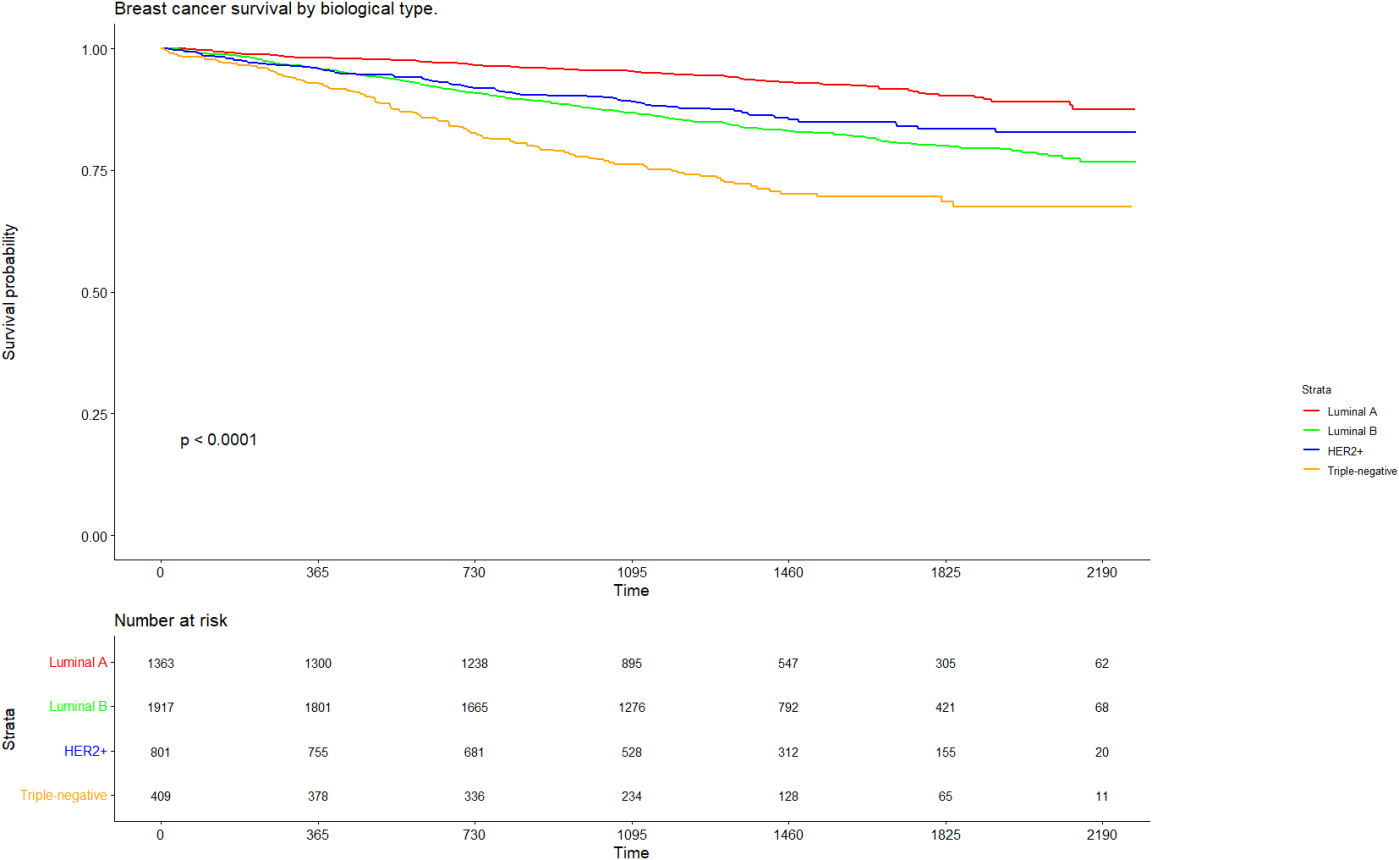
Breast cancer survival by biological type.

Patients with the luminal A subtype demonstrated the most favourable prognosis, with a 60-month Kaplan-Meier extrapolated survival rate of 90.3% (95% CI: 88.1%–92.5%). Conversely, those with triple-negative breast cancer had the poorest outcomes, with extrapolated survival declining to 68.5% (CI 95% 63.4%–74.0%) at 60 months. (**Figure 2**).

The 12-, 36- and 60 -month Kaplan-Meier survival rates among patients aged 50–69 years who participated in the screening program at DCOPiH were 99.6%, 94.8% and 89.5% respectively, representing about 3–5 percentage point increase compared to patients in the same age group who did not undergo screening within the pilot program (97.1%, 90.9% and 86.3%). Although the difference in survival rates appears clinically relevant, the Cox proportional hazards model indicated that the reduction in mortality risk did not reach conventional levels of statistical significance. The hazard ratio for the “DCOPiH Screening” group compared to the reference group (“No Screening in DCOPiH”) was 0.66 (CI 0.42-1.02 p = 0.059).

### Cancer stage and type – detailed survival analysis

**Figure 3:**
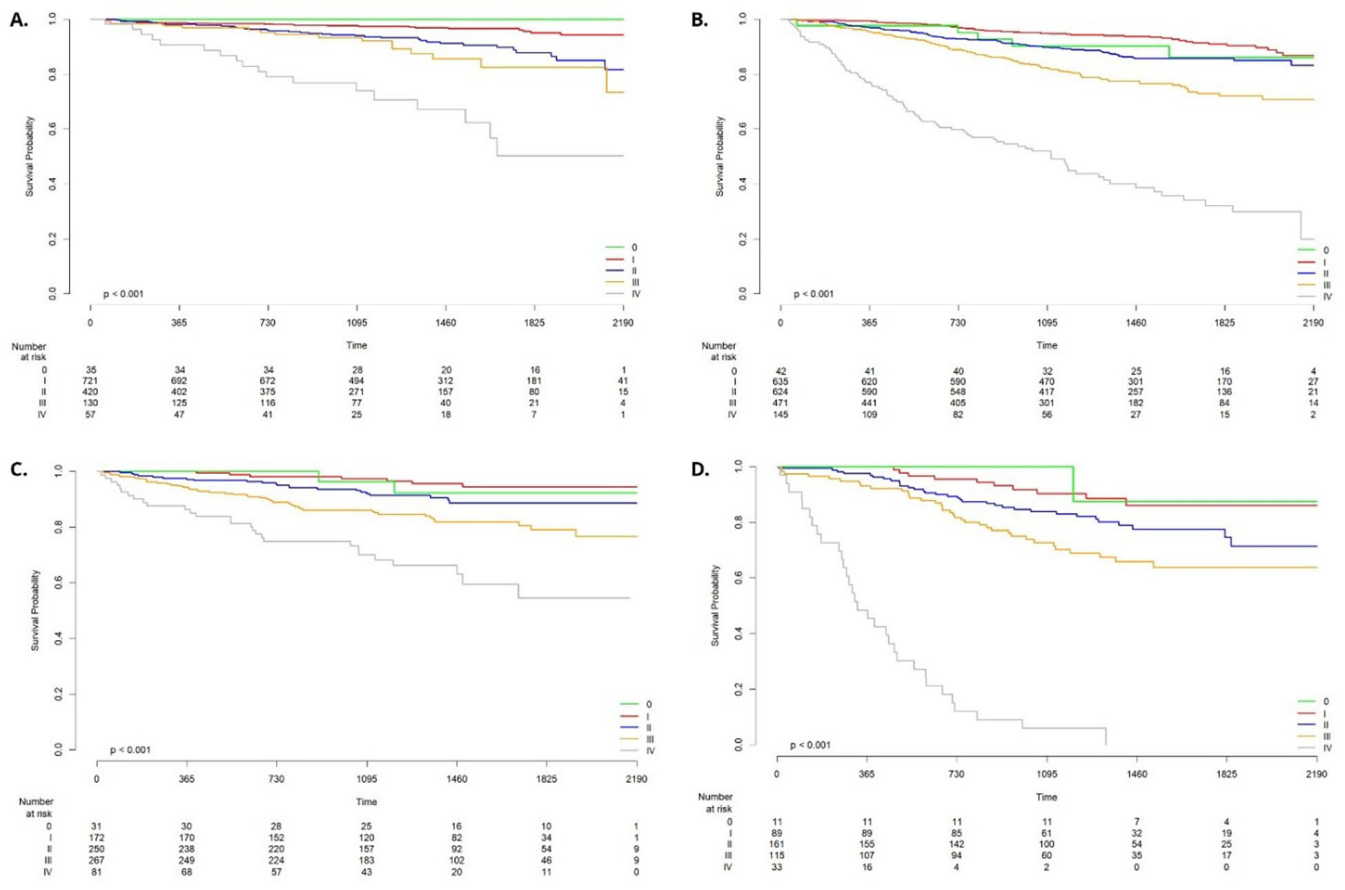
Breast cancer survival by stage for biological type Luminal A **(A)**, Luminal B **(B)**, HER2+ **(C)**, Triple negative **(D)**.

Analysis of the collected data confirmed that patients with the luminal A subtype exhibit the most favorable prognosis, although survival outcomes vary substantially depending on disease stage. The 60-month survival rate among patients with stage I and II disease was 95.1% and 87.8%, respectively. In contrast, survival decreased in more advanced stages, reaching 82.6% for stage III and 50.4% for stage IV. No death was recorded during the study in small subgroup of patients with stage 0 (n=35). Stage I was defined as the reference category in the Cox proportional hazards model, which demonstrated statistically significant differences in survival for stages II, III, and IV compared to stage I (p < 0.001) (**Figure 3A**).

Patients with the luminal B subtype showed a less favorable prognosis. The 60-month survival rates for stage I and II disease were 91.0% and 85.7%, respectively, while for stage III and IV, survival declined to 72.1% and 32.1%. In the small subset of patients diagnosed with stage 0 cancer (n=42) the 60-month survival was 86.1%. Using stage I as the reference, the Cox model indicated significantly worse outcomes for stages II–IV (p < 0.001 to p=0.002) (**Figure 3B**).

Among patients with the HER2-positive subtype, survival outcomes were intermediate. The 60-month survival rates were 94.4% for stage I and 88.7% for stage II. In more advanced stages, survival decreased to 79.1% for stage III and 63.0% for stage IV. The 60-month survival rate for stage 0 was 92.2%; however, this estimate is based on a small group (n = 31), with only two deaths reported during the study period. The Cox model confirmed significantly worse survival in stages III and IV compared to stage I (p < 0.001) (**Figure 3C**).

Among the analyzed cohort, patients with the triple-negative breast cancer subtype exhibited the poorest prognosis, with survival outcomes varying markedly by disease stage. The 60-month survival rates for stages 0, I, and II were 87.5%, 86.0%, and 74.6%, respectively. A further decline in survival was observed for patients diagnosed at stage III (63.8%), with a dramatic drop for those at stage IV, where 36-month survival reached only 6.1% and none of the patients observed survived 48 months (**Figure 3D**).

### Adjusted Cox analysis

The Cox proportional hazards model in all patients revealed no statistically significant differences in survival between stages 0 and I in total population – neither in unadjusted and adjusted analysis, as presented in **Table 3**. However, stages II, III and IV were associated with a significantly increased risk of death compared to stage I, with p-values <0.001, both in the unadjusted and adjusted calculations.

**Table 3:** Proportional hazard ratios for different cancer type, stage and age.

Variable		Unadjusted HRs– cancer type			Unadjusted HRs– cancer stage			Adjusted HRs – cancer type and stage			Adjusted HRs - cancer type, stage and age		
		HR	95% CI	P-value	HR	95% CI	P-value	HR	95% CI	P-value	HR	95% CI	P-value
Cancer type	Luminal A	1 (ref)	**-**	**-**	**-**	**-**	**-**	1 (ref)	**-**	**-**	1 (ref)	**-**	**-**
	Luminal B	2.45	1.94-3.08	<0.001	**-**	**-**	**-**	1.85	1.46, 2.34	<0.001	1.91	1.51, 2.41	<0.001
	HER2 positive	1.98	1.50-2.62	<0.001	**-**	**-**	**-**	1.20	0.90, 1.60	0.2	1.49	1.12, 1.98	0.007
	Triple negative	4.43	3.36-5.83	<0.001	**-**	**-**	**-**	3.53	2.67, 4.67	<0.001	4.15	3.13, 5.49	<0.001
Cancer stage	I	**-**	**-**	**-**	1 (ref)		1 (ref)	1 (ref)	**-**	**-**	1 (ref)	**-**	**-**
	0	**-**	**-**	**-**	1.13	0.55, 2.33	0.7	1.06	0.51, 2.19	0.9	1.02	0.49, 2.10	>0.9
	II	**-**	**-**	**-**	2.24	1.73, 2.90	<0.001	2.01	1.55, 2.60	<0.001	1.77	1.36, 2.30	<0.001
	III	**-**	**-**	**-**	4.13	3.22, 5.31	<0.001	3.55	2.75, 4.59	<0.001	3.31	2.55, 4.28	<0.001
	IV	**-**	**-**	**-**	14.4	11.1, 18.7	<0.001	13.9	10.7, 18.1	<0.001	12.4	9.52, 16.1	<0.001
Age group	60-69	**-**	**-**	**-**	**-**	**-**	**-**	**-**	**-**	**-**	1 (ref)	**-**	**-**
	<_30	**-**	**-**	**-**	**-**	**-**	**-**	**-**	**-**	**-**	1.14	0.47, 2.77	0.8
	30-39	**-**	**-**	**-**	**-**	**-**	**-**	**-**	**-**	**-**	0.56	0.37, 0.86	0.008
	40-49	**-**	**-**	**-**	**-**	**-**	**-**	**-**	**-**	**-**	0.43	0.30, 0.61	<0.001
	50-59	**-**	**-**	**-**	**-**	**-**	**-**	**-**	**-**	**-**	0.60	0.46, 0.79	<0.001
	70-79	**-**	**-**	**-**	**-**	**-**	**-**	**-**	**-**	**-**	1.57	1.27, 1.94	<0.001
	80+	**-**	**-**	**-**	**-**	**-**	**-**	**-**	**-**	**-**	3.53	2.83, 4.41	<0.001

CI, Confidence Interval; HR, Hazard Ratio.

In this analysis stage I was selected as the reference because there were more observations in this stage than in stage 0 for each type of cancer. In a similar vein, the 60–69 age group was selected as the reference point for age analysis, as it constituted the largest age stratum, thereby enhancing the statistical inference.

When examining the interaction between biological subtype and disease stage (**Tables 4**, **5**), a consistent trend emerged: survival was strongly dependent on disease stage across all subtypes. The triple-negative subtype showed the widest variation in survival by stage with the hazard ratio for stage IV in TNBC compared to stage I of 32.3 (15.6-67.0). In all subtypes no significant differences were observed between stages 0 and I, and in HER2-positive and triple negative no differences were also observed between stage II and I. Stages III and IV were consistently associated with significantly worse outcomes (p < 0.001).

**Table 4:** Overall survival – Cox proportional hazard ratios for disease stages within biological subtypes.

		Stage				
		0	I	II	III	IV
Molecular type	Luminal A	-^a^	1 (ref)	2.78 (1.66-4.67) <0.001	3.99(2.09-7.61) <0.001	12.8 (6.96-23.7) <0.001
	Luminal B	1.50 (0.60-3.77) 0.4	1 (ref)	1.77 (1.23-2.54) 0.002	3.31 (2.35-4.68) <0.001	12.8 (8.93-18.2) <0.001
	HER2-positive	1.50 (0.31-7.22) 0.6	1 (ref)	2.28 (0.97-5.33) 0.058	4.49 (2.03-9.95) <0.001	10.7 (4.65-24.4) <0.001
	Triple-negative	0.70 (0.09-5.46) 0.7	1 (ref)	1.95 (0.96-3.96) 0.064	3.23 (1.60-6.50) 0.001	32.3 (15.6-67.0) <0.001
	ALL	1.18 (0.55-2.33) 0.7	1 (ref)	2.24 (1.73-2.90) <0.001	4.13 (3.22-5.31) <0.001	14.4 (11.1-18.7) <0.001

No events were recorded among the patients with Luminal A stage 0 disease.

**Table 5:** Overall survival – Cox proportional hazard ratios for biological subtypes within disease stages.

		Biological type			
		Luminal A	Luminal B	HER2-positive	Triple-negative
Stage	0	–	–	–	–
	I	1 (ref)	2.14 (1.31-3.49) 0.002	1.27 (0.54-2.94) 0.6	3.45 (1.65-7.23) <0.001
	II	1 (ref)	1.40 (0.94-2.07) 0.10	1.03 (0.61-1.75) 0.9	2.46 (1.53-3.94) <0.001
	III	1 (ref)	1.86 (1.08-3.20) 0.025	1.45 (0.81-2.60) 0.2	2.86 (1.56-5.22) <0.001
	IV	1 (ref)	2.18 (1.31-3.63) 0.003	1.09 (0.60-1.97) 0.8	7.30 (4.05-13.2) <0.001
	ALL	1 (ref)	2.45 (1.94-3.08) <0.001	1.98 (1.50-2.62) <0.001	4.43 (3.36-5.83) <0.001

Hazard ratios for stage 0 could not be calculated due to no events in the Luminal A reference group.

## Discussion

This study provides comprehensive population-based insights into breast cancer survival in the Lower Silesia region during the pilot phase of the National Oncology Network (NON), with a specific focus on the prognostic relevance of clinical stage and biological subtype. The results confirm that disease stage at diagnosis remains the strongest determinant of patient outcomes, regardless of tumour biology. These findings align with international evidence and underscore the critical need for early detection, structured care coordination, and standardized reporting.

The share of categories 0+I (combined) fluctuated between 37.2% and 41.7% across the years with the share of stage I within a range of 34.5% to 41.2%. The lowest proportion was recorded in 2022 (37.2%) due to COVID19 pandemia impact, while the highest was in 2023 (41.7%). Overall, the average for the five-year period stands at 39.6%, showing a relatively stable but slightly increasing trend toward the end of the observed period. In contrast, the share of categories III+IV (combined) remained fairly stable, ranging between 27.3% and 29.9%. The highest proportions were observed in 2020 and 2022 (29.9%), whereas the lowest appeared in 2023 (27.3%). The five-year average is 28.9%, with no clear upward or downward trend overall. During the pandemic, from March 2020 to May 2022, the number of tests statistically decreased dramatically to an average of 37.93 per month for overall newly diagnosed breast cancer in DCOPIH, while positive diagnoses dropped to 24.74 per month. In comparison, from January 2018 to February 2020, the number of histopathological tests for breast cancer was relatively stable, averaging 67.23 per month, with 31.50 positive diagnoses (with positive diagnoses during this period was 46.85%) (8).

Consistent with previous studies, patients with the luminal A subtype exhibited the most favourable prognosis, with 60-month survival rates exceeding 90% in total population and 95% in stages 0 and I. This outcome reflects the relatively indolent clinical course and strong responsiveness to endocrine therapy characteristic of this subtype (9, 10). Luminal B tumours, although also hormone receptor–positive, were associated with a moderately worse prognosis—especially at advanced stages—likely due to higher proliferation indices and reduced endocrine sensitivity (11). Among HER2-positive patients, survival was high in early stages but declined at stage IV (54.6%), probably due the availability of targeted therapies such as trastuzumab and pertuzumab (12, 13). Triple-negative breast cancer (TNBC) was associated with the poorest prognosis, showing a precipitous drop in 60-month survival from 86.0-87.5% at stages 0 and I to only 63.8% at stage III. In stage IV TNBC cancer no patient was observed to achieve a 48-month survival and the study documented a 36-month survival rate of only 6.1% (14, 15). The fact that this particular type of cancer has such a low survival rate is especially notable given that this specific subtype comprised the highest proportion of patients in the youngest age group, i.e. those under 30 years of age.

The pattens of age structure within each biological subtype in our study reflect the known association between tumour biology and patient age, with hormone receptor–positive subtypes being more common in older women, and aggressive subtypes such as TNBC and HER2-positive tumours being relatively more prevalent among younger patients within their respective subtype distributions. Age at diagnosis may then inform risk stratification, surveillance intensity, and treatment planning.

Importantly, while biological subtype stratifies risk within stages, survival declined consistently across all subtypes with increasing disease stage. Patients with stage 0 and I disease had 60-month survival rates exceeding 86%, while those diagnosed at stage IV had survival rates as low as 0–54.6%, depending on subtype. Notably TNBC cases showed the most pronounced stage-dependent decline. These findings reflect the biological aggressiveness and limited treatment options for TNBC and reinforce the need for intensified systemic therapy and rapid intervention in this group.

Despite significant improvements in breast cancer treatment, Poland continues to lag behind many EU countries in survival outcomes. In 2021, breast cancer mortality in Poland was 20 per 100, 000 women - 5% higher than the EU average. While the mortality rate declined by 8% in Poland between 2011 and 2021, the EU average saw a 16% reduction during the same period. Moreover, while two-thirds of EU countries had reached five-year survival rates of ~80% for breast cancer patients diagnosed between 2000 and 2007, Poland only achieved a similar benchmark for patients diagnosed between 2015 and 2018 (16). For this period, five-year survival in Poland was 77%, compared to the EU average of 82%. Regional disparities further complicate the landscape: five-year survival in the 2010–2014 period ranged from 69.7% (Podkarpackie) to 79.8% (Mazowieckie), with Lower Silesia at 77.3%—broadly consistent with our observed outcomes.

The pilot implementation of the National Oncology Network in Lower Silesia aimed to address these systemic deficiencies. It introduced standardized diagnostic and treatment pathways, mandatory participation of multi-level care providers (SOLO I–III), and centralized coordination by Provincial Coordinating Centers. Additionally, it was the first nationwide program to implement real-time digital data collection and reporting for oncology quality indicators. The Lower Silesia Oncology Center was also the first in Poland to obtain iPAAC certification in breast cancer care, contributing to consistent and standardized data collection.

Nevertheless, the results reveal persistent gaps in early detection. Only 2.7% of patients in this population were diagnosed at stage 0, but European guidelines recommending that at least 15% of new breast cancer cases should be diagnosed at this stage in this population (17). In the pilot population for the Lower Silesia 7.3% had *in situ* breast cancer. Furthermore, only 36.0% of cases were detected at stage I, while stages III and IV accounted for 21.9% and 7.0%, respectively. These distributions stand in contrast to results from Norway (2005–2010), where 90.6% of cancers were diagnosed at stages I–II, and just 9.4% at stages III–IV, as well as similar benchmarks in the Netherlands and Spain (18).

These observations are particularly concerning given that participation in screening programs in Poland remains low - only 37% of eligible women underwent screening in 2023. In our analysis, patients aged 50–69 who participated in screening at DCOPiH had 36 and 60-month survival rates of 94.8% and 89.5 respectively, compared to 90.9% and 86.3% among non-participants. Although the difference did not reach conventional statistical significance (HR = 0.66 CI 0.42-1.02 p = 0.059), it reflects a clinically relevant benefit and aligns with previous European data showing that population-based screening programs reduce the incidence of advanced breast cancer and improve survival (19–24).

Indeed, systematic reviews and cohort studies from the Netherlands, Sweden, Finland, Italy, Germany, and France have consistently demonstrated that organized screening programs lead to a measurable reduction in the incidence of advanced-stage disease (T2+, N+, stage II+) (25). For example, Paci et al. (26) reported a risk ratio of 0.72 for stage II+ cancers in screened versus unscreened populations, while Dutch studies (27) showed annual reductions in advanced breast cancer incidence ratios ranging from 0.86 to 0.72 (19, 21). Our findings are consistent with these trends and reinforce the need for broader access and adherence to screening programs in Poland. Finally, while our results generally correspond to international registry data (e.g., Canadian Cancer Registry), notable differences remain in the distribution of disease stages and biological subtypes. For example, Canadian data showed about 47% of luminal A tumours, whereas this figure was 30.3% in our cohort (28). These discrepancies may reflect differences in population structure, access to care, and data completeness.

## Conclusions

Survival outcomes in breast cancer in Poland are strongly influenced by both the biological subtype and the stage at diagnosis. Early-stage detection through screening and rapid diagnostic pathways remain the most effective strategies to improve prognosis, particularly for aggressive subtypes such as TNBC and HER2-positive disease. The implementation of the National Oncology Network represents a step toward coordinated, data-driven cancer care in Poland, but further efforts are needed to increase screening participation, reduce diagnostic delays, and standardize treatment across regions.

## Data Availability

The original contributions presented in the study are included in the article/supplementary material. Further inquiries can be directed to the corresponding author.
