# Correction: Cancer survival in Lower Silesia: insights from a national oncology network pilot in Poland

**DOI:** 10.3389/fonc.2026.1820767

**Published:** 2026-04-08

**Authors:** Magdalena Władysiuk, Paweł Zawadzki, Paweł Predkiewicz, Robert Plisko, Dawid Błaszczyk, Rafał Matkowski, Adam Maciejczyk, Bozena Cybulska-Stopa

**Affiliations:** 1 Collegium Medicum, Chair of Epidemiology and Preventive Medicine, Jagiellonian University, Krakow, Poland; 2 Lower Silesian Oncology, Pulmonology and Hematology Center, Wrocław, Poland; 3 Department of Finance, Wroclaw University of Economics and Business, Wrocław, Poland; 4 HTA Consulting, Krakow, Poland; 5 Department of Oncology, Wrocław Medical University, Wrocław, Poland; 6 Department of Hematology and Oncology, Faculty of Medicine, Wroclaw University of Science and Technology, Wrocław, Poland

**Keywords:** biological subtype, breast cancer, national oncology network, outcomes, Poland, stage, survival

Affiliation “Department of Oncology, Wrocław Medical University, Wrocław, Poland" was omitted for author Rafał Matkowski. This affiliation has now been added for author Rafał Matkowski.

The original version of this article has been updated.

